# Synchronous vitellogenin expression and sexual maturation during migration are negatively correlated with juvenile hormone levels in *Mythimna separata*

**DOI:** 10.1038/srep33309

**Published:** 2016-09-15

**Authors:** Hai-Jun Xiao, Xiao-Wei Fu, Yong-Qiang Liu, Kong-Ming Wu

**Affiliations:** 1 State Key Laboratory for Biology of Plant Diseases and Insect Pests, Institute of Plant Protection, Chinese Academy of Agricultural Sciences, Beijing 100193, China; 2 Institute of Entomology, Jiangxi Agricultural University, Nanchang, 330045, China

## Abstract

Annual migration of pests between different seasonal habitats can lead to serious crop damage. Reproductive immaturity is generally associated with the migratory process (oogenesis-flight syndrome), but the mechanism of reproductive development during migration varies unpredictably. Here, the vitellogenin gene (*MsVg*) and three key regulatory enzyme genes (*MsJhamt, MsJheh* and *MsJhe*) related to juvenile hormone (JH) synthesis and degradation were identified and characterized in *Mythimna separata*. The relative expression of *MsVg* varied significantly in response to seasonal changes and was significantly correlated with stages of ovarian development. The relatively low levels of JH titer did not differ significantly in male moths but slightly increased in female adults during the migratory season, which was consistent with changes in mRNA levels for *MsJhamt, MsJheh* and *MsJhe*. JH titer was negatively associated with relative seasonal levels of vitellogenin mRNA transcripts and with ovarian development in migrating *M. separata*. The synchrony of *MsVg* expression with sexual maturation highlighted the potential of *MsVg* transcript levels to serve as an index to monitor the adult reproductive status. In addition, the level of JH and sexual maturity were correlated with the extent of JH in regulating the *MsVg* expression and reproduction during seasonal northern and southern migration.

The oriental armyworm *Mythimna separata* (Walker) (Lepidoptera: Noctuidae) is a serious migratory insect pest in Asia^1^ on Poaceae species such as *Zea mays* (maize), *Triticum aestivum* (wheat), and *Oryza sativa* (rice), and its outbreaks have caused large-scale crop losses in China over the last three years^2^. Seasonal outbreaks of this pest often occurs in northern and northeastern areas in warm climates. It cannot survive winter in northern China^1^, and thus in late autumn it migrates long distances to overwinter in the south^3^,^4^,^5^,^6^.

During migration, insects encounter a number of qualitative and quantitative trade-offs between migration and fecundity^7^. For example, the onset of reproduction may be delayed until after the migratory event^8^. Flight, such as long-distance migration, is accompanied with a cost of decreased fecundity, because nutritional reserves are shared between flight and reproduction, resulting in the ‘oogenesis–flight syndrome’^7^. The consequence of this syndrome in insects with limited nutritional reserves that are partly diverted into flight, often leads to reduced reproductive potential and at least a delay in the initiation of fecundity^8^,^9^.

This syndrome, however, is unlikely to be universal. For example, among overseas-migrating adults of *M. separata* captured in the early summer, both ovarian development and gravid females have been found, and migrating females were often found to have mated at least once^10^. Mating may occur during migration since oogenesis is switched on before oviposition during migration^10^,^11^.

Reproductive development during migration may be regulated by genes that are related to juvenile hormone (JH) synthesis and degradation and are responsive to vitellogenin gene expression and sexual maturation, with JH promoting egg production and provisioning^12^. Juvenile hormone is of great importance throughout the lifespan of insects; it acts to prevent metamorphosis during larval molting, and then regulates reproductive maturation^13^,^14^. In the final steps of JH biosynthesis, juvenile hormone acid *O*-methyltransferase (JHAMT) catalyzes the conversion of farnesoic acid (FA) and JH acid to their methyl esters, methyl farnesoate (MF) and JH^15^. A dynamic balance in JH titer in maintained through the regulation of JH biosynthesis in coordination with the regulation of JH degradation in the hemolymph and other peripheral tissues. JH esterase (JHE) and JH epoxide hydrolase (JHEH) are involved in the first step of JH degradation, in which juvenile hormone esterase (JHE) converts JHs to JH acid in a reversible reaction^16^. Juvenile hormone epoxide hydrolases (JHEH) irreversibly converts JH to JH diol. Previous research on JH degradation enzymes has mainly focused on JHE, JHEH has been less studied, and few *Jhamt* genes have been cloned; so little is known about *Jhamt* involvement in JH biosynthesis^15^,^17^,^18^,^19^,^20^,^21^,^22^,^23^.

In most insects, vitellogenin, the major nutrient reserve of the embryo and a precursor of the major egg yolk protein, is synthesized in the fat body, secreted into the hemolymph, and then incorporated into the developing oocytes^24^. Transcript levels for the vitellogen (*Vg*) can provide an indirect view of the dynamics of ovarian development; however, the molecular basis of *Vg* expression and JH synthesis in promoting adult reproduction during migration still has not been fully elucidated^25^.

The hormonal control of vitellogenesis varies widely among insect species. The juvenile hormone (JH) pathway interacts with key developmental/physiological pathways during oogenesis, especially in response to seasonal environmental conditions, flight and nutritional status^26^,^27^,^28^. For example, lepidopteran insects require either JH or 20-hydroxyecdysone (20E) to initiate Vg synthesis in the fat body^28^. Along with various neuroendocrine hormones, 20E also has gonadotropic roles. In most species, JH promotes Vg synthesis^29^, and in the grasshopper *Melanoplus sanguinipes*, JH titer increases after a long flight^30^; this increase may be an important component of migratory-enhanced reproduction. Either one of these hormones alone or in combination can regulate this process in wing-polymorphic crickets^26^. Regulation of reproductive vitellogenesis in conjunction with migration also comes with trade-offs related to the oogenesis–flight syndrome. For example, in wing-polymorphic insect species with a dispersal-capable morph, the dispersal ability comes at the expense of reproduction in long-winged morphs^31^. *Mythimna separata* is similar to other migrants in that JH inhibits migration and induces female reproduction^27^,^32^.

To further explore the physiological and molecular mechanisms underlying the reproductive changes during long-distance migration, we isolated cDNA sequences of JH acid methyltransferase (JHAMT, the final step regulatory enzyme for JH synthesis^16^,^17^), JH esterase (JHE, reversibly converts JH to JH acid^16^,^33^), and JH epoxide hydrolase (JHEH, irreversibly hydrolyzes JH to JH diol^16^,^34^). We hypothesized that there should be a correlation among the JH titer, mRNA expression of genes involved in JH synthesis and metabolism, which in return should induce vitellogenin synthesis and sexual maturity during long, seasonal migrations. The JH titer and a differential display analysis of the mRNA transcript levels for genes of enzymes in JH synthesis and metabolism were analyzed. We also used the relative amount of *MsVg* expression as an index of ovarian development to support a model of sexual maturation synchronous with migration or the oogenesis-flight syndrome, which are highly adaptive processes in migratory biology.

## Results

### Vitellogenin gene and seasonal expression

The full-length cDNA of the *M. separata* vitellogenin gene (*MsVg*) was 5699 bp, including an in-frame stop codon 92 bases upstream from the first ATG, an open reading frame (ORF) of 5280 bp (93-5372), which encoded a polypeptide of 1760 amino acids with a predicted molecular mass of 201.5  kDa and a theoretical isoelectric point of 8.81 and a consensus sequence directing polyadenylation 298 bases upstream from the poly A sequence (Figure S1). The MsVg protein contains a signal peptide of 16 amino acids (VSS-GR). A conserved domain search of the NCBI database revealed that the MsVg contains a sequence similar to conserved motifs such as the vitellogenin-N-terminal lipid binding domain (LPD_N) (29-724), RVRR cleavage signal (354-357), DUF1943 (757-1035), and C-terminal von Willebrand factor type D domain (VWD) (1418-1505) found in several other insects (Figure S1). A protein–BLAST (blastp) search showed that MsVg has high similarity to Vg isolated from species within the same family; it had 77% identity and 88% similarity to *Helicoverpa armigera* vitellogenin (HaVg, AGL08685).

**Figure 1:**
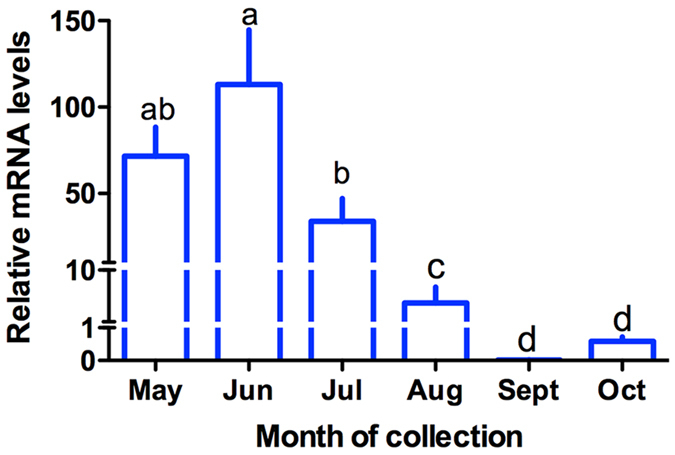
Mean (±SE) relative abundance of mRNA transcripts for the vitellogenin gene (*MsVg*) in *Mythimna separata*. Data were normalized relative to transcript levels for reference genes β-actin and GAPDH. Means with the same letters did not differ significantly at the 5% level as determined by Bonferroni test.

The relative expression levels of *MsVg* in the seasonal migratory female indicate that the mRNA level varied significantly in response to the collection date (ANOVA, *F* = 9.49, *d.f.* = 5,35, *P* < 0.0001), with high expression in early summer (higher in May and highest in June). Expression levels declined significantly after the peak in June, continuing to decline until nearly absent in September and October (Fig. 1).

**Figure 2:**
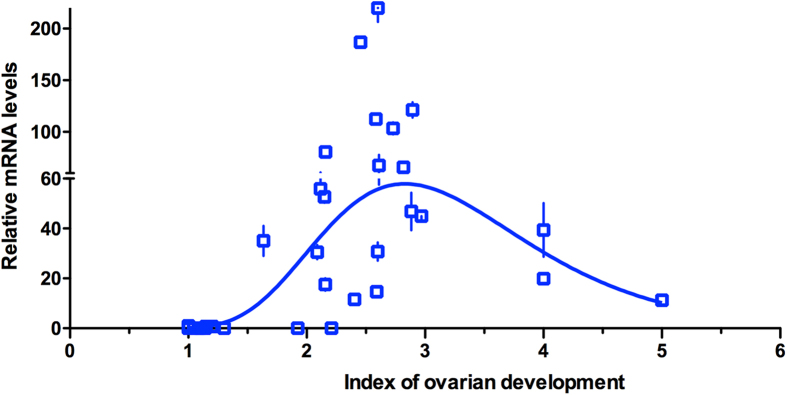
Relationship between relative level of *MsVg* expression and index of ovarian development in seasonal-migratory female adults of *Mythimna separata* captured in a vertically pointed searchlight trap on Beihuang Island in the Bohai Gulf of China in 2012.

Correlation of the relationship between the index of ovarian development (level 1 to 5: 1, no follicular differentiation; 2, distinct follicular development; 3, developed ovaries with some chorionated eggs; 4, some eggs laid; 5, more than 50% of the egg complement laid^10^) and the relative mRNA transcript level of the vitellogenin gene (*MsVg*) showed a Gaussian distribution. Transcripts were barely detectable during early ovarian development (level 1), increasing as the ovaries developed (level 2 to 3), peaking when the ovaries were nearly fully mature (level 3, calculated result = 2.83), then decreasing as the eggs were laid (level 4 to 5) (Fig. 2).

### Seasonal changes in JH titer

**Figure 3:**
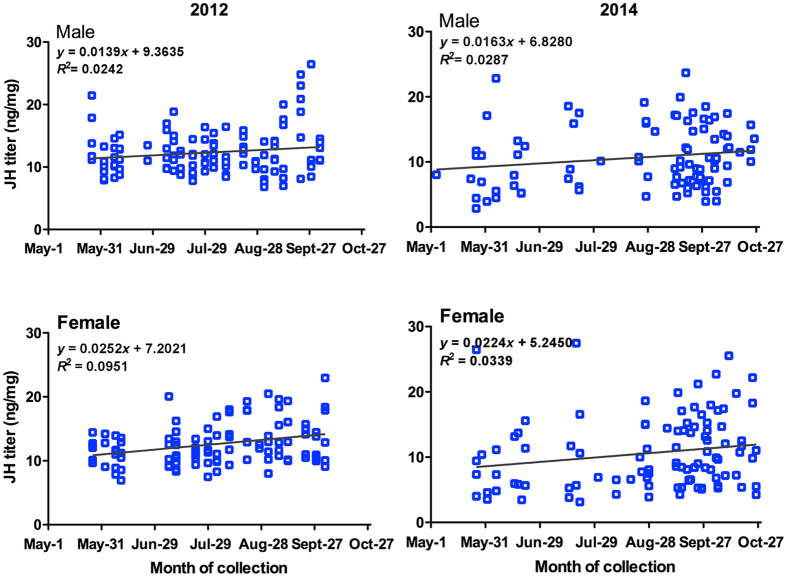
Seasonal changes in juvenile hormone (JH) titer in migrating adults of *Mythimna separata*, captured by a vertically pointed searchlight trap on Beihuang Island in the Bohai Gulf of China in 2012 and 2014.

The JH titers in migrating adults over the entire season differed substantially among individuals, ranging from a daily average of 6.82 to 26.46 ng/mg in 2012 and 5.21 to 19.14 ng/mg in 2014 for male moths and from 6.92 to 22.92 ng/mg in 2012 and 3.47 to 25.53 ng/mg in 2014 for female moths (Fig. 3). Seasonal JH titer in male moths did not differ significantly, but a slight increase was found in females during autumn in the migratory season (Male: *F* = 2.828, *P* = 0.0953, *R*^2^ = 0.0242 and Female: *F* = 11.14, *P* = 0.0012, *R*^2^ = 0.0951 in 2012; Male: *F* = 2.629, *P* = 0.1084, *R*^2^ = 0.0287 and Female: *F* = 3.487, *P* = 0.0651, *R*^2^ = 0.0339 in 2014) (Fig. 3). Linear regression analyses revealed that JH titer was negatively correlated with *MsVg* mRNA transcript levels and the index of ovarian development (Figure S2).

### *MsJhamt* cDNA and seasonal expression

The full-length nucleotide sequence of the cDNA (1730 bp; hereafter designated as *MsJhamt*) obtained by a combination of modified 5′ and 3′ RACE revealed a predicted ORF of 849 bp encoding 283 amino acid residues, with a calculated molecular mass of 33.275 kDa and a calculated pI value of 5.48. The cDNA has a short 5′ UTR (119 bp) and a relatively long 3′ UTR (762 bp), which encompasses the initial cDNA sequence (Figure S3). A search for conserved domains in the NCBI database revealed that the predicted ORF contained a sequence similar to conserved motifs found in several SAM-dependent methyltransferases.

The deduced protein sequence of *MsJhamt* showed 78% identity to the *H. armigera* Jhamt (BAF63630), 68% identity to the *S. litura* Jhamt (BAF63629), 56% identity to the *Samia ricini* Jhamt (ABE98256) and *B. mori* Jhamt (NP-001036901), and 52% identity to the *D. plexippus* Jhamt (EHJ73944).

**Figure 4:**
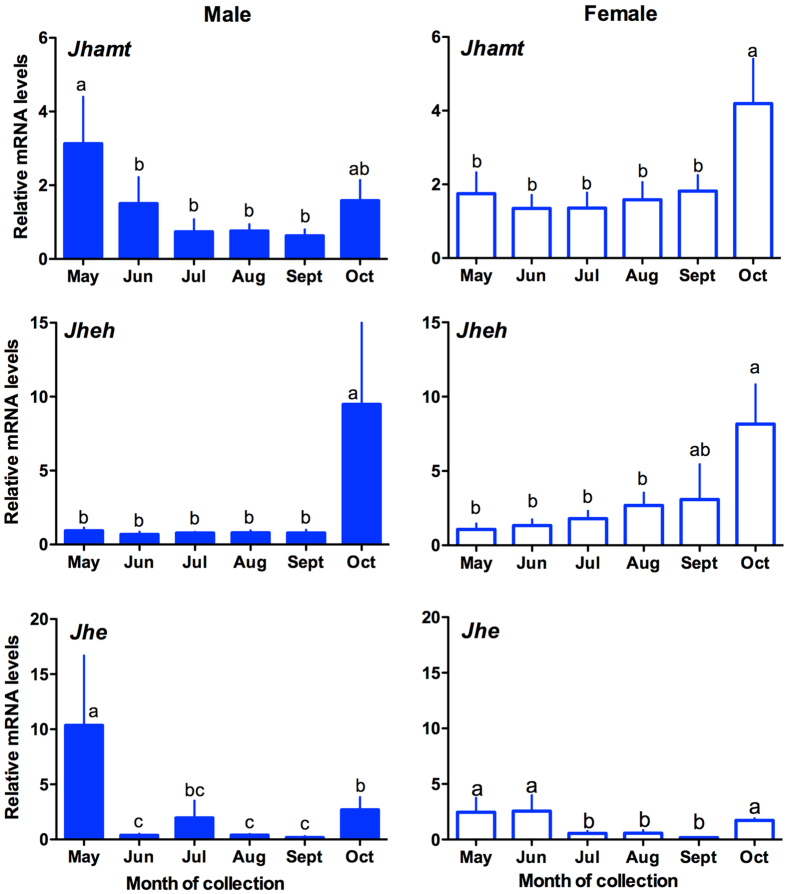
qRT-PCR analysis of mRNA transcript levels for JH acid methyltransferase (*Jhamt*), JH epoxide hydrolase (*Jheh*) and JH esterase (*Jhe*) in migrating adults of *Mythimna separata* from May to October in 2012. Data were normalized relative to transcript levels for reference genes for β-actin and GAPDH. Bars indicate the standard error of six replicates; means with the same letters did not differ significantly at the 5% level as determined by a Bonferroni test.

The relative mRNA expression patterns of *MsJhamt* were investigated using RT-qPCR in the adults of both sexes of *M. separata* during their seasonal migration. Although abundant *MsJhamt* mRNA was detected in both males and females, expression levels were relatively low and differed between the two sexes (Fig. 4). In the males, significantly higher expression was recorded in May than in those captured from July to September (*F* = 3.15, *d.f.* = 5,31, *P* = 0.0156). However, in female moths, only an acute increase in October was detected in the expression of *MsJhamt (F* = 2.81, *d.f.* = 5,31, *P* = 0.0331).

### *MsJheh* cDNA and seasonal expression

We obtained a full-length cDNA encoding Jheh in *M. separata (MsJheh*), which comprised 1509 bp with an open reading frame of 1383 bp encoding 461 amino acid residues. The deduced protein without the putative signal peptide had a theoretical molecular mass of 52.758 kDa and a calculated pI value of 8.56. *MsJheh* contained three active site residues (Asp227, Glu402, and His429) that make up the catalytic triad, Tyr290, Tyr372, and the HGXP motif, which serve as part of the oxyanion hole of epoxide hydrolases (EHs) (Figure S4).

Multiple sequence alignment of this gene with other sequences suggests that *MsJheh* shares extensive structural features with the epoxide hydrolases (EHs); with the domains containing the catalytic triad and the oxyanion hole, especially from insect JHEHs. A blastp search revealed that the predicted amino acid sequence encoded by *MsJheh* had the highest amino acid similarity (99%) and identity (83%) to *Spodoptera exigua* Jheh (ABD85119). *MsJheh* also had 93–95% amino acid similarity and 62–65% identity to *H. armigera* Jheh (ACM78602), *B. mori* Jheh (NP001037201), and *D. plexippus* Jheh (EHJ74147).

The seasonal expression pattern of *MsJheh* in migratory moths of *M. separata* revealed that expression in adults captured in October was significantly higher than in moths captured from May to August (Male: *F* = 2.69, *d.f.* = 5,30, *P* = 0.0327; Female: *F* = 2.96, *d.f.* = 5,31, *P* = 0.0268) (Fig. 4).

### *MsJhe* cDNA and seasonal expression

The cDNA sequence of *MsJhe* had an open reading frame of 1752 bp and 583 deduced amino acids, with a calculated molecular mass of 63.503 kDa and a calculated pI value of 6.09. The five catalytic motifs known to be essential for the catalytic activity of JHE and other esterases were very well conserved in *MsJhe*. The deduced amino acid sequence encoded by *MsJhe* (Figure S5) showed 64% identity to a sequence from the cotton bollworm, *H. armigera* Jhe (AEB77712) and *Heliothis viriplaca* Jhe (AGB93712), 59% identity to *S. litura* Jhe (ACV60229), 52% identity to *B. mori* Jhe (AAR37335) and 48% identity to the *D. plexippus* Jhe (EHJ63342).

The pattern of *MsJhe* mRNA expression was also investigated in both male and female moths (Fig. 4). In males, *MsJhe* expression peaked in May (*F* = 17.02, *d.f.* = 5,31, *P* = 0.0001, Bonferroni test: *P* = 0.0307 between May and October; *P* = 0.0266–0.0357 in the paired Bonferroni comparison of October with June, August and September). The *MsJhe* expression in female adults captured in May, June and October was significantly higher than in females captured from July to September (*F* = 1.77, *d.f.* = 5,31, *P* = 0.0490, Bonferroni test, *P* = 0.001–0.048 in the paired Bonferroni comparison of October with July, August and September).

Linear regression analyses revealed that the seasonal JH titer was positively correlated with mRNA transcript levels for *Jhamt, Jheh* and *Jhe*, except for *Jhe* in female moths (Figure S5).

## Discussion

### JH titer and mRNA levels for JH-regulating enzymes during migration

In general, a decrease in JH synthesis associated with an increase in JH degradation will lower the JH titer^13^,^16^, and increased synthesis with decreased degradation will raise the JH titer. In a linear regression analysis between the monthly JH titer as the dependent variable and the transcript level of *Jhamt, Jheh* or *Jhe* as an independent variable, the seasonal JH titer was positively correlated with mRNA transcript levels for *Jhamt, Jheh* and *Jhe* (Figure S6), except for *Jhe* in female moths. Therefore, JH levels were relatively low during the entire seasonal migration, mainly from decreased synthesis of JH and the upregulation of *Jheh* and *Jhe,* which led to an increase in JH degradation.

Although the correlation analysis between seasonal JH levels and relative levels of mRNA transcripts of *Jhamt, Jheh* and *Jhe* during seasonal migration may reflect the role of JH in regulating reproduction, the study has some limitations. First, we examined only three key genes from the JH biosynthesis and degradation pathway. Second, the spatiotemporal expression patterns of these three genes were not investigated. On the basis of studies on other species^16^,^29^,^35^,^36^,^37^,^38^,^39^,^40^,^41^, we assumed that these three enzymes would be good indicators of JH metabolism. Although the results cannot fully explain the mechanism by which JH regulates reproduction during seasonal migration, they form a basis for further study using RNA interference, RNA sequencing and related techniques to gain insight into the role of JH in regulating reproduction during seasonal migration in *M. separata.*

### Juvenile hormone and vitellogenesis and sexual maturation

In the current study, seasonal JH titer was negatively correlated with mRNA transcript levels for *MsVg* and the index of ovarian development (Figure S2). The seemingly contradictory correlation between JH titer and sexual maturity and *MsVg* expression can be easily interpreted. Lower JH titer is associated with higher mRNA transcript levels for *MsVg* and sexual maturity in early summer. At the collecting site for the adults on Beihuang Island, which is close to the destination of the northern migration, a large proportion of the migrating females captured in early summer were sexually mature, some were gravid, and some had even mated^10^. When the seasonal migration is almost finished and sexual development almost completed, a higher JH titer is unnecessary and should not be a restriction at this stage. In autumn, increasing JH titer may be essential to restart ovarian development in adults with low Vg expression and immature ovaries during the long overseas migration to the south, and during the next migration, both ovarian development and mating may occur^10^. A slight increase in JH titer during autumn migration may thus be the switch to turn on sexual development before the insects reach their destination in the next few days.

Vg synthesis may also depend on various neurosecretory hormones from the brain or 20-hydroxyecdysone (20E) in most insects^14^,^35^. Lepidopteran insects require either JH or 20E to initiate Vg synthesis in the fat body^28^. Juvenile hormone regulation of Vg synthesis is known in several insects; increasing JH titer is always associated with increasing vitellogenin gene expression^12^,^13^,^16^. In the burying beetle *Nicrophorus orbicollis*, mRNA transcripts for Vg, Vg concentrations in the hemolymph, ovarian maturity and JH titer increase in parallel during sexual maturation^36^. In the red flour beetle *Tribolium castaneum,* ecdysteroids, not JH, regulate ovarian growth and primary oocyte maturation^35^. In mosquitoes, although both JH and 20E are essential for oogenesis, 20E is more important for regulating vitellogenesis^37^.

Whether the migratory female lays some proportion of eggs at a suitable site along the migration route or does not lay any eggs until arriving at its destination is not well understood^38^. When the monarch butterfly *D. plexippus* migrates from eastern North American to overwinter in central Mexico, the adults are in reproductive diapause. During this journey, if given the suitable conditions, females will break diapause and lay eggs. It is possible that these females continue flying southward to Mexico because the reproductive status and migratory status are not tightly coupled^39^. In the spring, the monarch butterflies mate and then fly northward to lay eggs^40^. In contrast to recent claims about the oogenesis-flight syndrome, many studies have revealed that this phenomenon is not universal among migratory noctuid moths; some adults appear to be exclusively pre-reproductive during migration^10^,^11^,^41^,^42^. The migratory behavior persists after reproductive maturation and mating in *M. separata* is often observed. In fact, in the early summer, almost 100% of the migrating females captured on Beihuang Island were sexually mature (oocyte development was complete; >level 3) and had mated at least once^10^. In the current study, the synchronicity of *MsVg* expression with the level of sexual maturation indicated that the seasonal dynamics of *MsVg* expression may serve as a candidate index to monitor ovarian development during the seasonal migration of *M. separata.*

### *Vg* gene expression during migration

Synthesis of Vg, the yolk precursor, is correlated with ovarian maturation because Vg is the main source of nutrition for the embryo^24^, and is thus also an important factor for population proliferation. In oviparous females, Vg synthesis is an important step in ovarian maturation and oocyte development. In *Apis mellifera*, the total Vg that accumulated in 3 days comprises 70% of all proteins expressed, and this high production is maintained throughout the adult life^43^.

The increase in the number of immature female moths of *M. separata*, starting in late August indicates the initiation of the fall migration to the southwest^6^,^10^. Although long-distance migration, presumed to be a costly behavior, may result in a trade-off between adult reproduction, including ovarian development, and the energy needed to construct the adult flight apparatus and to fuel flight^11^,^44^, other examples show that the reproduction-flight trade-off effect may be overestimated^31^,^45^. Nevertheless, migratory flight is a bet-hedging evolutionary life history strategy for colonizing of habitats that are unpredictable or distant^38^. Southern-migrating adults of the oriental armyworm *M. separata*, which make up 74.68% of the entire population, might need to fly a long distance to a particular site where their offspring can overwinter^6^. A higher level of *MsVg* expression in early summer may shorten the pre-oviposition period after the moth arrives at its northern destination, and the low expression of *MsVg* in late autumn may ensure the success of the next long-distance southern migration.

Further analysis of the key regulatory elements in hormonal metabolic pathways during seasonal migration of the devastating pest *M. separata* will help us elucidate the regulatory mechanism controlling reproduction during seasonal long-distance migration. Also, the current work may be useful for developing control strategies, for example, identifying potential targets for RNAi applications for pest control^22^.

## Methods

### Insects

Overseas-migrating adults of *Mythimna separata,* were all captured by a vertically pointed searchlight trap on Beihuang Island in the Bohai Gulf of China (38°23′200′′N, 120°54′500′′E)^6^. To minimize the effect of any diel periodicity in JH titer and mRNA expression, measurements were made from adults collected between 6:00 and 8:00 am. The insects were then directly frozen in liquid nitrogen and stored at −80 °C until use.

Randomly chosen female moths collected on a given day were dissected to determine their reproductive status based on the ovarian developmental status detailed in previous studies^6^,^10^. The sum of individual levels of ovarian development divided by the number of dissected female moths was used to calculate average daily indexes of ovarian development. *MsVg* expression in female adults of *M. separata* captured in traps as aforementioned during early May to mid-October 2012 was quantified by RT-qPCR. A Gaussian regression distribution model was used to analyze the correlation between *MsVg* expression and the index of ovarian development.

### JH titer determination

JH was extracted using a hexane modification of a described protocol^30^,^32^,^46^,^47^. In detail, after wings were removed and fresh mass was weighed, moths were homogenized in 1 mL ice-cold methanol ether (1:1, v/v), and then 2 mL ice-cold hexane was added to extract the JH. The homogenate was allowed to settle at room temperature for 20 min. After centrifugation at 12,000 rpm at 4 °C for 10 min, the supernatant was collected and transferred to another 10 mL glass vial and kept on ice. The extraction was repeated three times, and the supernatants were combined. The extracts were dried under a nitrogen stream and then re-dissolved in methanol for analysis using methanol–water (80:20, v/v; 0.8 mL/min) as the mobile phase in an Agilent chromatograph (Santa Clara, California, USA) equipped with a Zorbax SiOH C18 column (4.6 mm × 250 mm) and a UV detector (218 nm). JH standards were used to establish the standard line for the peak area. JH titers were quantified by comparing JH peak area with the quantitative standards line. For JH titer determination, the solvents were all HPLC grade from Fisher Scientific (Fairlawn, NJ), SciTech (Czech) or Sigma (St. Louis, MO).

### cDNA cloning and sequencing

Total RNA was extracted from the collected adults using the Trizol reagent (Invitrogen, Carlsbad, CA, USA). After RNA was treated with DNase I (Invitrogen), first strand cDNA was synthesized using the SuperScript III Reverse Transcriptase system (Invitrogen). The quality of the cDNA templates was determined using the primers in Table S1, designed according to the nucleotide sequence of the *M. separata* actin gene (GenBank GQ856238) with the following PCR program: 4 min at 94 °C; 40 cycles of 30 s at 94 °C, 30 s at 55 °C, and 30 s at 72 °C; 10 min at 72 °C.

Degenerate primers were designed according to known genes from other insects and used to amplify the partial mRNA sequence of *MsVg, MsJhamt, MsJheh* and *MsJhe*. Degenerate primers (GF1, GF2/GR1) for *MsVg MsJhamt, MsJheh* and *MsJhe* and gene-specific primers *MsVg*-F1/GR2 (Table S1) were designed to amplify a fragment of the partial mRNA sequence by nested PCR. Cycling conditions were as follows: 94 °C for 4 min; 40 cycles of 94 °C for 30 s, 55 °C for 30 s, 72 °C for 2 min; 10 min at 72 °C. RACE method was used to amplify the 3′ and 5′ ends with a SMART RACE cDNA Amplification Kit (Clontech, Palo Alto, CA, USA) and specific primers (Table S1) according to the manufacturer’s protocol. To ensure that the 5′/3′ fragments were from the same gene, specific primers containing the full ORFs were designed according to the 5′/3′ sequences of untranslated regions, which were used to amplify the entire ORF sequences with the pfu enzyme (Table S1). The PCR cycling conditions were as follows: 94 °C for 4 min; 40 cycles of 94 °C for 30 s, 55 °C for 30 s and extension of 1 min/kb at 72 °C for each gene; 10 min at 72 °C. All of the PCR products were purified and cloned into a cloning vector (pEASY-T, TransGen, Beijing, China), then inserted into *Escherichia coli* DH5α; and finally the clones were sequenced. The 3′ end sequence containing the poly(A) tail was obtained from several clones. For *MsVg*, the 5′ RACE products amplified with primer *MsVg*5R1, yielded multiple bands ranging from 0.5 to 2.0 kb in an agarose gel. The PCR product was subcloned into pEASY-T and sequenced. The 5′ end was confirmed using a nested 5′ RACE assay and another primer *MsVg*5R2; the product, a single band (≈1.6 kb) in an agarose gel was subcloned into pEASY-T and sequenced. Several of the longest clones contained an identical sequence, suggesting that it is the 5′ end sequence of the transcript. Finally, the sequences obtained from the initial FDD fragment, the 5′ and 3′ RACE products, and the RT-PCR products were combined to generate a full-length sequence of *MsVg*. The signal peptide of *MsVg* was analyzed using the SignalP 4.1 Server (http://www.cbs.dtu.dk/services/SignalP/) and had the characteristics present in most Vgs. The sequence reported in this paper was deposited in the GenBank database (accession KF501044 for *MsVg* cDNA, KM926339 for *MsJhamt*, KM926340 for *MsJheh* and KM926341 for *MsJhe*).

### Quantitative real-time PCR (qRT-PCR)

The transcripts of *MsVg, MsJhamt, MsJheh* and *MsJhe* were quantified using a 7500 Fast Real-time PCR System (Applied Biosystems, Carlsbad, CA, USA). Total RNA was extracted from the whole body of *M. separata* and subjected to the recommended DNase step. Quality and concentration of the resulting RNA samples were measured using a Nanodrop spectrophotometer (Thermo Fisher Scientific). An equal amount of RNA was transcribed in subsequent cDNA synthesis utilizing the SuperScript III Reverse Transcriptase system (Invitrogen) and oligo(dT) primer following the manufacturer’s protocol, and RT-qPCR reactions were performed using the gene-specific primers (as shown in Table S1). RT-qPCR was carried out with the TaqMan method in 20 μL reaction volume comprising 1 μL of template cDNA (200 ng), 10 μL of 2*Premix Ex Taq (Takara, Japan), 0.4 μL of each primer (10 μM), 0.4 μL of Rox References Dye, and 0.8 μL probe (10 μM) and 7 μL of sterilized H_2_O. Thermal cycling conditions were 95 °C for 15 min and 40 cycles of 95 °C for 15 s and 60 °C for 34 s. After PCR, an automated melting curve analysis demonstrated the specificity of the PCR product, as displayed by a single peak. Each RT-qPCR reaction was done with three technical replicates for each cDNA sample. β-actin (GQ856238) and glyceraldehyde-3-phosphate dehydrogenase (GAPDH, HM055756) were used as internal genes for normalization in the RT-q-PCR analysis. The amount of *MsVg, MsJhamt, MsJheh* and *MsJhe* mRNA was calculated using the 2^−ΔΔCT^-quantification method^48^. At least six independent replicates were used.

### Statistical analyses

Data were statistically analyzed using STATA 9.0 Version (StataCorp LP, College Station, Texas, USA). Means were compared using ANOVA and multiple comparisons by Bonferroni tests, with a significance level of alpha = 0.05 for all comparisons. Values are presented as the mean ± SE. The generalized linear model (GLM) was used to analyze the relationship between monthly JH titer as the dependent variable and the mRNA transcript levels of *Jhamt, Jheh* and *Jhe* as the independent variable. The correlations among either the levels of *MsVg* mRNA transcript level or the index of ovarian development as the dependent variable and monthly average JH titer as the independent variable were also analyzed by GLM.

## Additional Information

**How to cite this article**: Xiao, H.-J. *et al*. Synchronous vitellogenin expression and sexual maturation during migration are negatively correlated with juvenile hormone levels in *Mythimna separata. Sci. Rep.* **6**, 33309; doi: 10.1038/srep33309 (2016).

## Supplementary Material

Supplementary Information
