# Supplementary material for: Synchronous vitellogenin expression and sexual maturation during migration are negatively correlated with juvenile hormone levels in Mythimna separata
Source: Sci Rep. 2016 Sep 15;6:33309. doi: 10.1038/srep33309 (PMC5024112; doi:10.1038/srep33309)
Supplement: Supplementary Information [file srep33309-s1.pdf]

Synchronous vitellogenin expression and sexual maturation during migration are negatively correlated with juvenile hormone levels in *Mythimna separata*

Hai-Jun Xiao[1,2], Xiao-Wei Fu[1], Yong-Qiang Liu[1], Kong-Ming Wu[1]

[1] State Key Laboratory for Biology of Plant Diseases and Insect Pests, Institute of Plant Protection, Chinese Academy of Agricultural Sciences, Beijing 100193, China

[2] Institute of Entomology, Jiangxi Agricultural University, Nanchang, 330045, China

Table S1. Primers used in this series of experiments.

Primer name	Nucleotide sequence (5' to 3')
**Detection of quality of RNA samples and cDNA templates**	
**Actin-F**	CATCTACGAGGGTTACGC
**Actin-R**	CATCTGTTGGAAGGTGGA
***MsVg, MsJhamt, MsJheh* and *MsJhe* cDNA amplification by DNA walking and RACE method**	
**VgGF1**	TTYARRAARATGGARACTGA
**VgGF2**	AAYCCYAAYAAYATGCC
**VgGR1**	TSRGCCATWKCYTGCATCAT
**MsVgF2**	GCTGCTATCCTGAACTTATT
**VgGR2**	GWRCAGWANGGYTGRTAYTG
**MsVg5'R1**	GGCGTTCCTGCTGTTGGACCTCTGT
**MsVg5'R2**	CACCTGGGCTGCTTCCTCACCTTGT
**MsVg3'F**	TCTACACGCCCTTCAGCACGGTTG
**Jhamt GF1**	AGCNTSCARAAGAGAGATGC
**Jhamt GF2**	GAYNTNGGHTSNGSNBHNGGW
**Jhamt GR1**	GATNTTRAAWGGRTTGATWGCT
**MsJhamt 5'R1**	TCCCCGTCTTCGTTCAGCAGGTTG
**MsJhamt 3'F**	TACAACCTGCTGAACGAAGACGGGGAG
**MsJhamt orf F**	GCAAGCAGTGGTATCAACGC
**MsJhamt orf R**	TGGGATTGGCTCCTTTCAGA
**Jheh GF1**	GGATGGCCVGGYTCCGT
**Jheh GR1**	GYTCRAACGCCAGGAARTG
**MsJheh 5'R1**	TTAGTAAACCTCAGGGCGAGTCCACCG
**MsJheh 3'F**	CGGTGGACTCGCCCTGAGGTTTACT
**MsJheh orf F**	GAGTCCAGTGTGTTGTGTGA
**MsJheh orfR**	AAACTTTAATAACCCCTAACC
**Jhe GF1**	GRRGTCATBARATTCTTCAT
**Jhe GR1**	YGTSATCACDTTTAATTACAG
**MsJhe 5'R1**	CTTTGCCAAGTCCGCATGGATTCC
**MsJhe 5'R2**	TGCGGAACGTCTCACACTCAGCACT
**MsJhe 3'F**	TCCATGCGGACTTGGCAAAGGAGAT
**MsJhe orf F**	CGGATCCACAGCCTACTGAT
**MsJhe orfR**	GAAACCAGCACAACCACACC
**Race UPM**	CTAATACGACTCACTATAGGGCAAGCAGTGGTATCAACGCAGAGT
**Race NUP**	AAGCAGTGGTATCAACGCAGAGT
**Real-time RT-PCR**	
**MsVg RT-F**	TGCTGAGGTCCCGCTTCT
**MsVg RT-R**	CCGTCGCGGATGTTCAAC
**MsVg RT probe**	CAGTACTACACCCAAGCCGAAGGCATCC
**MsJhamt F**	GCAACAACAAGTGGAGCAAGTG
**MsJhamt R**	TCGACTTCTTTTTCAGGGTCCTT
**MsJhamt RT probe**	TCGACCGATACGTGTCGCCCTATCA
**MsJheh F**	TGCTCGACGATAAGATGGTTCA

**MsJheh R**	TTCTACTTGCTAGATGTTCCTCGACTA
**MsJheh RT probe**	CGGCGCTTTCTTCCCATCCCTC
**MsJhe F**	ACTGGGTGCAGAGGAATGCT
**MsJhe R**	CTGCCCCGCGATCGT
**MsJhe RT probe**	AACTTCGGCGGAGACCCCAATGA
**Actin –RT-F**	TCTCGCTGCCGTAGAGTCAA
**Actin –RT-R**	CGCACATTTTGGCGTTTGT
**Actin RT probe**	ATCCACCGCGTCCCAAACCAAA
**Gapdh–RT-F**	TCAAGGGCACCGTTGACA
**Gapdh–RT-R**	GGAGAGGACGGAGATTTTGTTG
**Gapdh RT probe**	CAAGATGGCTTCCTCGTCGTCAACG

```
1                                                                                                GT
3     ACCCCTATAAAGACTGTGATATTTAAATACATTTCATTGACATCTCTATCAAACGATCTTAAAAATATACGTAGAATTTGCTAATAAACC
93    ATGTCATTGCTAATACCATGTTTCTTTGCAGCCGTCGTCTCCTCAGGGAGACTGAGCGAACAGCAGTTGGATAACCAATGGCCCTGGCAA
1      M  S  L  L  I  P  C  F  F  A  A  V  V  S  S  G  R  L  S  E  Q  Q  L  D  N  Q  W  P  W  Q
183   ACTGGAAGACTATACCGCTATGACGTCAACACCCACACCCTGGCCCGTCTTCAGGAAGGCGCCAGCACTGGCAACGCTTTCAGGGCCAAG
31     T  G  R  L  Y  R  Y  D  V  N  T  H  T  L  A  R  L  Q  E  G  A  S  T  G  N  A  F  R  A  K
273   TTCATCCTCCGTGTCGTGTCCCCAGGCCGCCTTCAGGCTAGGCTGGAAAACCCCCAACGGGCCCAGTTCCACCAGCAGCTGCCCAACAAC
61     F  I  L  R  V  V  S  P  G  R  L  Q  A  R  L  E  N  P  Q  R  A  Q  F  H  Q  Q  L  P  N  N
363   ATGGCCATCCCTTCGGACCTCAAATACGAAACTGTGCAGAACTTGGACAAACCTTTCGAGATCTCCGTTGAAGGTGGTCGCGTCCTCGGC
91     M  A  I  P  S  D  L  K  Y  E  T  V  Q  N  L  D  K  P  F  E  I  S  V  E  G  G  R  V  L  G
453   CTCAATCTGCCATCAGTCTTCCAGCTCTCTCACGAGAACTTGCTGAAGGGTCTGATCAGCGCGCTGCAAGTCGACTTATCTGCTTACCAC
121    L  N  L  P  S  V  F  Q  L  S  H  E  N  L  L  K  G  L  I  S  A  L  Q  V  D  L  S  A  Y  H
543   CATGTGCGCAACCTACCGAACAACTTCGACAGGGAACGCCAGCAGGGTCTTTTCAAAAAGATGGAGGCTGACGTCACTGGTGACTGCGAA
151    H  V  R  N  L  P  N  N  F  D  R  E  R  Q  Q  G  L  F  K  K  M  E  A  D  V  T  G  D  C  E
633   ACCATGTACACAGTCTCTCCTGTGGCCGCTGAATGGCGACGAGAGCTGCCGTTGTTCGTTTCAGAAGAAGACCCCATGGAGATCACCAAG
181    T  M  Y  T  V  S  P  V  A  A  E  W  R  R  E  L  P  L  F  V  S  E  E  D  P  M  E  I  T  K
723   AGCAAGAACTACGGCCACTGCCACCACCGTGTCGCTTACCACTTCGGAGTACCCGAAGGTTTTGAATGGACAGGAACCGCACACAGCAAT
211    S  K  N  Y  G  H  C  H  H  R  V  A  Y  H  F  G  V  P  E  G  F  E  W  T  G  T  A  H  S  N
813   GAAGAGAAGCAATTCATCTCGCACTCTGCCACCTCCCGCATTCTTGCTGGTAAGCAGGGTCCTATCTACAAGGCTGAAACCACGAGTTTC
241    E  E  K  Q  F  I  S  H  S  A  T  S  R  I  L  A  G  K  Q  G  P  I  Y  K  A  E  T  T  S  F
903   GTGACCGTGCACCCCCACTTATATGGCCAGCAGAAAGCGCAGGTACACAGCTACGTAAGCTTGAACTTAATGTCCGTTGAACAAGATAGT
271    V  T  V  H  P  H  L  Y  G  Q  Q  K  A  Q  V  H  S  Y  V  S  L  N  L  M  S  V  E  Q  D  S
993   GGTGCCGAATGGCAAAAACCTGAAGGCAGCCGTCAAATCCACACTCTTCTGTACGCAATGAGCACCAAACAAATGGCTTACCACGACCAG
301    G  A  E  W  Q  K  P  E  G  S  R  Q  I  H  T  L  L  Y  A  M  S  T  K  Q  M  A  Y  H  D  Q
1083  TCTTCAAAGTCAGGCGAGTCTGCTGAATCCCATGAGCACATAAATGTGCAATTGGACGGTCTGAGAAGCAGAGTGCGCCGTTCTGATGTT
331    S  S  K  S  G  E  S  A  E  S  H  E  H  I  N  V  Q  L  D  G  L  R  S [R  V  R  R] S  D  V
1173  CAACAAGAGCGAATGTTCAACAAGGACTGGAGGTCGTCGAGTTCCAGTGACTCTTCATCAGCTTACATCAACGATGACGTTCCCAGGATG
361    Q  Q  E  R  M  F  N  K  D  W  R  S  S  S  S  S  D  S  S  S  A  Y  I  N  D  D  V  P  R  M
1263  AATGAGCCAGCATACGCTGCTCTCTACATGAGTGCTCTTCCGCGTGGTGATAAGAAACAAAACGTGTTGAACGTCCAGAAGCTGCTCCAG
391    N  E  P  A  Y  A  A  L  Y  M  S  A  L  P  R  G  D  K  K  Q  N  V  L  N  V  Q  K  L  L  Q
1353  GACATTGCTCAACAACTGCAAAACCCCAACAACATGCCTAAAGCTGACTTTTTATCCAAGTTCAACATCCTCGTCCGCATTGTTGCTTCC
421    D  I  A  Q  Q  L  Q  N  P  N  N  M  P  K  A  D  F  L  S  K  F  N  I  L  V  R  I  V  A  S
1443  ATGAGCACTGAACAGCTCGCCCAGATCAGCCGTGGCATTGAAGTCGGCAGAAATTCAAACAACAGTGTTAAAACTGATATGTGGATGATC
451    M  S  T  E  Q  L  A  Q  I  S  R  G  I  E  V  G  R  N  S  N  N  S  V  K  T  D  M  W  M  I
1533  TTCCGTGATGTTGTCGTACAAGCTGGCACACCCCCGGCCTTTACGCAAATTAAGATTTGGATTATGAGCAAGAAGTTACAAGGTGAGGAA
481    F  R  D  V  V  V  Q  A  G  T  P  P  A  F  T  Q  I  K  I  W  I  M  S  K  K  L  Q  G  E  E
1623  GCAGCCCAGGTGCTGTCCACTTTGGCACGTACCCTTCGTTACCCCACCAAGGAAATTATGGCACAGTTCTTCGAGCTCGCCATGAGCACA
511    A  A  Q  V  L  S  T  L  A  R  T  L  R  Y  P  T  K  E  I  M  A  Q  F  F  E  L  A  M  S  T
1713  GAGGTCCAACAGCAGGAACGCCTTAACACTAGTGCTTTGATCGCTGCAACGGAATTCATTCGCATGGGTCAAGTCAACAACGAAACTGCT
541    E  V  Q  Q  Q  E  R  L  N  T  S  A  L  I  A  A  T  E  F  I  R  M  G  Q  V  N  N  E  T  A
1803  CACTCTTACTACCCCACTCACATGTATGGCCGTCTTGCCCGCAGACACGACAGATTCGTGCTGGACAACATCCTTCCTCGTCTCTCCGAA
571    H  S  Y  Y  P  T  H  M  Y  G  R  L  A  R  R  H  D  R  F  V  L  D  N  I  L  P  R  L  S  E
1893  TTGTTGAAACAGGCTATTGAGGCTAAAGAATGGAGCAGGGCTCTGGTGTACGTTAAGGCTATTGGCAACTTAGGACACCCTGAGATCTTA
601    L  L  K  Q  A  I  E  A  K  E  W  S  R  A  L  V  Y  V  K  A  I  G  N  L  G  H  P  E  I  L
1983  TACGTATTCTCTCCTTACCTGGAGGGTCGTATTGAAGTGTCTACACACCTCCGCGTTCAAATGATTGTAAATCTTCGCCATCTCTCGAAC
631    Y  V  F  S  P  Y  L  E  G  R  I  E  V  S  T  H  L  R  V  Q  M  I  V  N  L  R  H  L  S  N
2073  CAGAAGGATAAATACGTGCGTGCTGTGCTCTACAGCATCATGAGGAACACTGCCGAGCCATACGAAGTGCGAGTGGCTGCTATCCTGAAC
661    Q  K  D  K  Y  V  R  A  V  L  Y  S  I  M  R  N  T  A  E  P  Y  E  V  R  V  A  A  I  L  N
2163  TTATTCATGGCTCACCCGACCGCTGAGATGATGCAAGTTATGGCCCAGATGACCCATGATGATCCCAGTATCCAGGTTCGCGCTGTTCTC
691    L  F  M  A  H  P  T  A  E  M  M  Q  V  M  A  Q  M  T  H  D  D  P  S  I  Q  V  R  A  V  L
2253  AAGAATGGCATCTTAACCGCTGCCAGCTTGAAGGAACCTCGCTTTTGGCACCTGTCTAGGGCAGCTCAGTCTGTCAAGGAAATCGTAACA
721    K  N  G  I  L  T  A  A  S  L  K  E  P  R  F  W  H  L  S  R  A  A  Q  S  V  K  E  I  V  T
2343  CGAGAGAACCTTGGTATGCACTACTCTAACAAATTCTACATCAACAACTATGTTAATGATGGAGAACTGGGGAACTTCCAAGTAGCATCT
751    R  E  N  L  G  M  H  Y  S  N  K  F  Y  I  N  N  Y  V  N  D  G  E  L  G  N  F  Q  V  A  S
2433  TACATTGGTGGTGAAAGCAGTGTTCTGCCTACATACCAGAGACACTCCTGGATGAACAAACTCGGAGGAAGGGCCCTTGAAAACATGATT
781    Y  I  G  G  E  S  S  V  L  P  T  Y  Q  R  H  S  W  M  N  K  L  G  G  R  A  L  E  N  M  I
2523  GGCGCTTCATTCTCCGATGTGCAGGAAATCGTTGATTTCATCAAGACAATGCTGTTTGAGCCTCAGAGGTCTGAAGCCAACCACAAATTC
811    G  A  S  F  S  D  V  Q  E  I  V  D  F  I  K  T  M  L  F  E  P  Q  R  S  E  A  N  H  K  F
2613  AATGCCCAAAGAATTACGGAAATGCTGAACATCAAGCGTGAATCTCAAAGGCCACTCGAGGGCTCTTTCTTCTACGACATTTTCAACCAG
841    N  A  Q  R  I  T  E  M  L  N  I  K  R  E  S  Q  R  P  L  E  G  S  F  F  Y  D  I  F  N  Q
2703  GAGAGATTCTACAGTTTCGACGAAGGAGACCTTATCAGGCTGGTACAAGACATCATGGAGTACATGAAGGAGGTAGAACAAGGTGTCGAG
871    E  R  F  Y  S  F  D  E  G  D  L  I  R  L  V  Q  D  I  M  E  Y  M  K  E  V  E  Q  G  V  E
2793  AAGCACTACACCAAAGTGTTCAACTCCAACCAGGTTTCCGTTATGTTCCCCATCGCCTCAGGTATGCCTTTCATCTACAAATACAAGGAG
901    K  H  Y  T  K  V  F  N  S  N  Q  V  S  V  M  F  P  I  A  S  G  M  P  F  I  Y  K  Y  K  E
2883  CCTGTCGTTATGCACATCCAGAGCAAGGCTAAGGGCAAGATCGACCGTGACCCCAAGAACCGCAACAATCTGTCCTCTTTCATGGATAAG
931    P  V  V  M  H  I  Q  S  K  A  K  G  K  I  D  R  D  P  K  N  R  N  N  L  S  S  F  M  D  K
2973  GAACTCCAACTTACGCTTGCCAGAAACATCGATGGAACCGTTGGTTTCATGGACACCCTTACTAACCAGCTGGCTATTGCTGGAGTCGTC
961    E  L  Q  L  T  L  A  R  N  I  D  G  T  V  G  F  M  D  T  L  T  N  Q  L  A  I  A  G  V  V
3063  AAGAAATACCAGATTAACGTACCTGTCAAGCTGAGCTTCCAGATGGAATCTGGACAAGCTAAAATGAAGGTGGAACCTCTGCGCCCTGAC
991    K  K  Y  Q  I  N  V  P  V  K  L  S  F  Q  M  E  S  G  Q  A  K  M  K  V  E  P  L  R  P  D
3153  CAGGACTACACCATCGCTCATTACAGCGTTTGGCCGTTCACTACTTACCAGAAGAAGGATACTTTAGTGCCCTACTCTCAGGATCCTGCC
1021   Q  D  Y  T  I  A  H  Y  S  V  W  P  F  T  T  Y  Q  K  K  D  T  L  V  P  Y  S  Q  D  P  A
3243  ACCAAAATAGTAGAACGCTCAAGGAAGGTACTGTCCACTGACATGAAGTTCGGCCAACAAGCTGGCACTGTTTTCCAACTGCAAGGATAC
1051   T  K  I  V  E  R  S  R  K  V  L  S  T  D  M  K  F  G  Q  Q  A  G  T  V  F  Q  L  Q  G  Y
3333  TCCTACTCCACGGACTTCAGAAACGCTGGAAACCTGGTTCAGGCCCTGTACAGCATTGGTGACTTATTAGGTGCCCGTGACATCGCTCTG
1081   S  Y  S  T  D  F  R  N  A  G  N  L  V  Q  A  L  Y  S  I  G  D  L  L  G  A  R  D  I  A  L
3423  ACGCACTACAACTTCAGATATCTTGGAAAACAGTCGCAGAACAAGGTTGTCACCTTTACTGCTGCTCATGATACATTCTTCAACCAGAAA
1111   T  H  Y  N  F  R  Y  L  G  K  Q  S  Q  N  K  V  V  T  F  T  A  A  H  D  T  F  F  N  Q  K
```

```
3513 CAAAACGGAGAGCTGGGACAAGCTAACAACAGGAACGATGTGACGCCCAACAGCGGAGTCCGCCGTGAGGAGATGGTCAAGCGTGTTGCA
1141  Q  N  G  E  L  G  Q  A  N  N  R  N  D  V  T  P  N  S  G  V  R  R  E  E  M  V  K  R  V  A
3603 TCTGGAATCAACAATGCTAAAGTACAAGTTGTCGACGTTAGCGCCACATTCGAGGGCCCCCTGAAACAGGAGTACGTACTGACTGCCGCT
1171  S  G  I  N  N  A  K  V  Q  V  V  D  V  S  A  T  F  E  G  P  L  K  Q  E  Y  V  L  T  A  A
3693 ATCGCCAACAGCCCAGTCGACCGCAAAATCCAATATGTCCTCTTCGCTGGAAGGAACTCAATCAAGCTGGGTAACGAACAGATCAACGCT
1201  I  A  N  S  P  V  D  R  K  I  Q  Y  V  L  F  A  G  R  N  S  I  K  L  G  N  E  Q  I  N  A
3783 GTCGTCAAGGTAACAAAGCCCGAAATATCGCCACTGAACTTCTTGGAAGCTCTGCAAAAGGACATGAAGATGACTTACGAAGCCGACATC
1231  V  V  K  V  T  K  P  E  I  S  P  L  N  F  L  E  A  L  Q  K  D  M  K  M  T  Y  E  A  D  I
3873 AAGTTCGGTCAGAATGGAAACATTCACATCCAAGGAAACACTGAACGCTCTCAGAAGTTCACCGAACACCTGAAGAACCATCCTTTGGCT
1261  K  F  G  Q  N  G  N  I  H  I  Q  G  N  T  E  R  S  Q  K  F  T  E  H  L  K  N  H  P  L  A
3963 AAGTTGGTGCAGCAGGACATTGCTAATGGCAACCTGTACCAGGCTACTTCTCTCAAGATGCTGATTATGGCCCATACCCCAGATAACTTC
1291  K  L  V  Q  Q  D  I  A  N  G  N  L  Y  Q  A  T  S  L  K  M  L  I  M  A  H  T  P  D  N  F
4053 AAGGCGTCAGTGACCTACAAGAACATGAGCCCCATGTACATGTACTGGGCTGCCCAAGTGAACCAGATTCTGAAGCAACTGAGTTGGAAC
1321  K  A  S  V  T  Y  K  N  M  S  P  M  Y  M  Y  W  A  A  Q  V  N  Q  I  L  K  Q  L  S  W  N
4143 ACGGAAGTGAACCCATTGAAGAGGGTTGCTGATGGTAAACTGCAGTTGGAGGTTGAGACTTTCTACGGCGACAACACCCTTCGTTTCGAG
1351  T  E  V  N  P  L  K  R  V  A  D  G  K  L  Q  L  E  V  E  T  F  Y  G  D  N  T  L  R  F  E
4233 ATGACTTCACCATCCGGTTTAGTTCGTGTCGAGAACTTACCGATTCCCATGATCACTCCCTACATTGTGTCCGTCTACACGCCCTTCAGC
1381  M  T  S  P  S  G  L  V  R  V  E  N  L  P  I  P  M  I  T  P  Y  I  V  S  V  Y  T  P  F  S
4323 ACGGTTGAGCGCATCGTCAACTACCTGACCAGCTACCAGTACCAGCCTTTCTGTACCATTGACGGCACCAAGGTGAGGACCTTCAGCAAC
1411  T  V  E  R  I  V  N  Y  L  T  S  Y  Q  Y  Q  P  F  C  T  I  D  G  T  K  V  R  T  F  S  N
4413 CGCACCTACGACTACGAGCTGTCCCGTTCCTGGCACGCGGTGATGCAGGAGGAGTACAACAAGGCCCGCGGCAAGTGGGACGAGCTGGTC
1441  R  T  Y  D  Y  E  L  S  R  S  W  H  A  V  M  Q  E  E  Y  N  K  A  R  G  K  W  D  E  L  V
4503 ATCCTCGCGAGGAGACCCACCCAGCTGCAGCAGCAGATCTACATCTCTTACAAAACCGAAACTGGCAAGGACCTGGAAATCGAGATTTTA
1471  I  L  A  R  R  P  T  Q  L  Q  Q  Q  I  Y  I  S  Y  K  T  E  T  G  K  D  L  E  I  E  I  L
4593 CCGTCAAAGTCCCACAAGGCTATCGTTCAAGTAAAGACCAACAGCAAGAAGATCTCTGAGGGAGACTTGAGCATTTACTGGGATGATGTT
1501  P  S  K  S  H  K  A  I  V  Q  V  K  T  N  S  K  K  I  S  E  G  D  L  S  I  Y  W  D  D  V
4683 GCTGAGGTCCCGCTTCTGCAGTACTACACCCAAGCCGAAGGCATCCTGATGTTGAACATCCGCGACGGACGTCTCCGCCTCATGTACGAC
1531  A  E  V  P  L  L  Q  Y  Y  T  Q  A  E  G  I  L  M  L  N  I  R  D  G  R  L  R  L  M  Y  D
4773 GGCCAGCGCCTGGTGCTTACCACCCAAGACTACCGTACCACCACCAGAGGCATTTGCGGCCAGAACACCGGTGAGCCACGCAACGACTAC
1561  G  Q  R  L  V  L  T  T  Q  D  Y  R  T  T  T  R  G  I  C  G  Q  N  T  G  E  P  R  N  D  Y
4863 CTGACTCCTCATGGTTTGGTTGACCTGCCTCAGCACTATGGTGCATCCTTCGCCCTTGACGTCGAAGACAGCGACCCTAAGACACAAGGC
1591  L  T  P  H  G  L  V  D  L  P  Q  H  Y  G  A  S  F  A  L  D  V  E  D  S  D  P  K  T  Q  G
4953 CTTAAGATGGAAGCTCAGCAAAAGGCGTACCAGCGAATTCCTAAATACACCGCTATTCTCCGCTCCGATGAAGAATGGAACAAAGCCATC
1621  L  K  M  E  A  Q  Q  K  A  Y  Q  R  I  P  K  Y  T  A  I  L  R  S  D  E  E  W  N  K  A  I
5043 ATGCAGAGAGACCAGGACTGGGACTCCCAGAATGTTTACAGGACCAGAAGCCACGGTTTGACGGTTCGTGGACAGTGCCAGGTGCAGCAG
1651  M  Q  R  D  Q  D  W  D  S  Q  N  V  Y  R  T  R  S  H  G  L  T  V  R  G  Q  C  Q  V  Q  Q
5133 CAAATCCAGTACCATGAGAACCATGGGGAGATTTGCATTACCACCATCCCCCTGCCGTCTTGCCCGTCCCATTGCCATGGAGAAGGCTAC
1681  Q  I  Q  Y  H  E  N  H  G  E  I  C  I  T  T  I  P  L  P  S  C  P  S  H  C  H  G  E  G  Y
5223 AAGGTTCAAGCTGCTCAGGTCATTTGCCGGCCAAAGACTGACGAACAGTTCAGATCCTTTAGAAGCCAGATTCACCAGGGCCAGAACCCT
1711  K  V  Q  A  A  Q  V  I  C  R  P  K  T  D  E  Q  F  R  S  F  R  S  Q  I  H  Q  G  Q  N  P
5313 CAGGTTGATGGAGTTCCGAGAGTAGAACAGTACAGAGTACCCACGTCGTGCAAGGCGTAAGAACCCTTCCACTTACCACCTAATTCAATT
1741  Q  V  D  G  V  P  R  V  E  Q  Y  R  V  P  T  S  C  K  A  *
5403 AACTACTTAGATATTAGTAGAAAATAATGGCATTGAACTTGGACCACGAATATTTTATTAATAATAAACTATGTGTTCATATTTACTTTG
5493 GCAATATAATATTTATATATTTCGAAATTTAGTGTCGTATTATGTTATTATTACTATGACATTTATTTATGTATAAAAATATTTATTTTA
5583 TATAAGTATTTTATGTACCTCTTTGTATTTATCTATAAAGACTATAATGTATTATTATTGCAGTAGAATAGTTTGTAAAGAGCAAGTGAA
5673 AAAAAAAAAAAAAAAAAAAAAAAAAA
```

Figure S1. The nucleotide and deduced amino acid sequence of the vitellogenin cDNA of *M. separata*. The line shows the position of the signal peptide. The conserved domain of Vg as LPD_N, DUF 1943 and von Willebrand factor type D domain (VWD) are shown in blue, yellow and green background, respectively. Residues highlighted by the box (□) are the RVRR cleavage sites. The conserved domain of DGQR RGICG, and polyserines are shown in purple background. The suggested start (ATG) and stop (TAA) codons are indicated in red background.

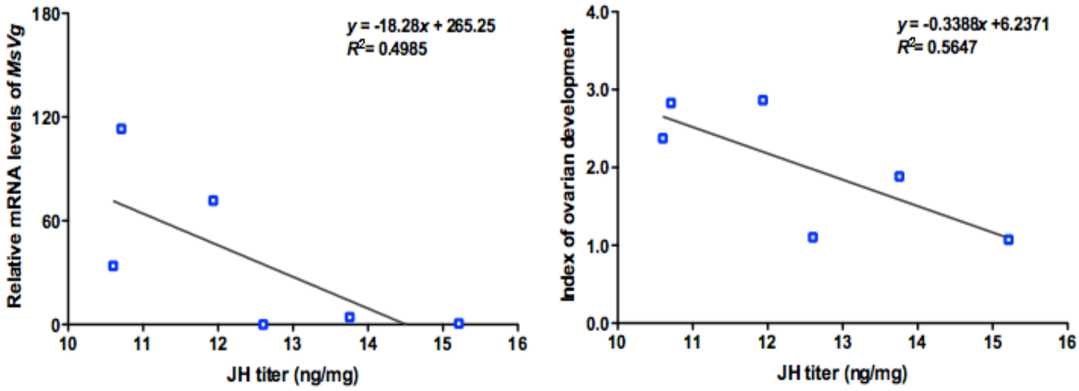

Figure S2. Linear regression analysis between the levels of *MsVg* mRNA transcript level or the index of ovarain development as dependent variable, and monthly average JH titer as independent variable in *M. separata*.

```
1     ACATGGGGGACTGTCCGCCGAAACAGCGCTCCCACACCACGCGTGCGCACCCAAAAATACTAAACATAAAATCATAAACACAAAAATCCT
91    CTGAAAATCCCTGCAACAAAAAAAAAATCATGAATAACGCTGTTTTATACGAAAAATGCAACAGCCTGCAGAAGAGAGATGCTCTTTTGTG
1                                 M  N  N  A  V  L  Y  E  K  C  N  S  L  Q  K  R  D  A  L  L C

181   TTTAGAAGAAAACGCGTCGAAAATGAAGTGGAAGAAAAACAATAACAATATCTTAGACATCGGCTGCGGAGATGGAAGCGTCACCAACAT
22     L  E  E  N  A  S  K  M  K  W  K  K  N  N  N  N  I  L  D  I  G  C  G  D  G  S  V  T  N  M

271   GCTGAAGAAGTACATCCCCACTGACTTCAAACTCCTCGGCTGCGACATCAGTGAGAAGATGGTGAACTTTGCCAATAATCACCACTGCAA
52     L  K  K  Y  I  P  T  D  F  K  L  L  G  C  D  I  S  E  K  M  V  N  F  A  N  N  H  H  C  N

361   TGAACAGACTTCCTTCACCGTTCTCGACATAGAAGGGGACCTGCCTGAAGGGATGAAGGGAAAATTCGACCACGTGTTTTCATTCTACGC
82     E  Q  T  S  F  T  V  L  D  I  E  G  D  L  P  E  G  M  K  G  K  F  D  H  V  F  S  F  Y  A

451   TTTGCACTGGATTATAAATCAAAAACGAGCATTCACCAATATCTACAACCTGCTGAACGAAGACGGGGAGTGCTTCATGATCTTCCTCGC
112    L  H  W  I  I  N  Q  K  R  A  F  T  N  I  Y  N  L  L  N  E  D  G  E  C  F  M  I  F  L  A

541   GGGTGCGCCTGTGTTCGACGTGTACCGGGTGCTGGCTCGCAACAACAAGTGGAGCAAGTGGCTGCAGAACGTCGACCGATACGTGTCGCC
142    G  A  P  V  F  D  V  Y  R  V  L  A  R  N  N  K  W  S  K  W  L  Q  N  V  D  R  Y  V  S  P

631   CTATCACGACTCTAAGGACCCTGAAAAAGAAGTCGAGAAAATGCTGGAAGAGATTGGTTTCGTTGACTACGATGTGCAGTGTAAAAACTC
172    Y  H  D  S  K  D  P  E  K  E  V  E  K  M  L  E  E  I  G  F  V  D  Y  D  V  Q  C  K  N  S

721   AGTGTACGTGTTTGAAGACTTACATGTGTTACGAAGTGCCTTCACAGCGGTAAATCCTTTCAAAATCCCTGAAGATAAGTTCGATGACTT
202    V  Y  V  F  E  D  L  H  V  L  R  S  A  F  T  A  V  N  P  F  K  I  P  E  D  K  F  D  D  F

811   CATGTCGGACTACTTGGACATACTGAAGGAATTGCAGATAATGACCAAGTTCAACAATAATTATGAAACAAGCGTTCAGTTCCGCTATCG
232    M  S  D  Y  L  D  I  L  K  E  L  Q  I  M  T  K  F  N  N  N  Y  E  T  S  V  Q  F  R  Y  R

901   TCTACTAGTGGTGTATGCTCGCAAACCAGCCTCACAGGATAAACTACTGGAAGAATTAAACAAGCAGGTCTGAACATCTATGCTGGAACC
262    L  L  V  V  Y  A  R  K  P  A  S  Q  D  K  L  L  E  E  L  N  K  Q  V  *

991   GAAAACTCTGTACTTTTAGTGCATTTGTTCGCCCATTTTGATTACCTACTTGCAGTTTATTTATTTTACATGCTTGAAAATTGGCCTATA
1081  GGAGAAGTAATGTTGTATATATGATATATCATATCCATAGTGATAAACATGGTCCTTCGAGCCGGATAGCAACAGAGAGTATAATCATTT
1171  TGTTTACATTATTATTGCTTAAAGTACCTAAAAGCTGAAAAGGAATATTGTCTGAAAGGAGCCAATCCCACCACGTTGCACATTTATAGT
1261  ATGTTCTCTTGTTTTGGTAGAAAAGAAGATTGTAATATCATGGTCCATTGTTTGATAACTTGGTTCGCATATTCGGAAGCTATATGTCAA
1351  TTAATTATTTGGAGTTTCAAAATAATCGTAATAACGAAAAAAATCATGTTGATCTCTGGTTGAATTTCTGTGCTGATTCTATAATTTTCT
1441  GTTTTATGGTGATGGCGATTGATACGTGTTTAAGGGGCTAGTAAATAAGAATGTGATGTTTTTTTTAGATGGTGACGATAATATTGTCTA
1531  TCGTCATTTTATGTAAATAGGTGGTATTTAAACTTTATATAATTATGATCTTTATATTAGTTGTTATTTATTTATATTGAATAGATTTTT
1621  AGAATAATAGTTGTAAGGGGTTACGGCAATATTCGATGAGCCTAAAGATGTAATTTTTATTAAAGATTGACTTACTTACCTAAAAAAAAA
1711  AAAAAAAAAAAAAAAAAAAA
```

Figure S3. Nucleotide and deduced amino acid sequence of *MsJhamt*. “□” stand for residues highlighted by the box are the conserved SAM binding motif (motif I). The positions of the primers used in the initial degenerate RT-PCR are shown by thin lines under the nucleotide sequence. The stop codon is indicated by an asterisk. The small red triangles indicate the residues in contact with SAM and substrates.

```
1       ACATGGGGGAGTCCAGTGTGTTGTGTGAACACGGGCAGACGTGGTAACATCGTCATGGGTTTCCTAGTAAAAGTGGTGTTGGTGGCCGCC
1                                                                       M  G  F  L  V  K  V  V  L  V  A  A
91      TTGGGCGTGGCAGTATGGTATTACTTCAACGGCTGCTGCAAGCAAACCATACCCAAGTTCGACAGTGAAGAGTGGTGGGGGCCAACAGCG
13       L  G  V  A  V  W  Y  Y  F  N  G  C  C  K  Q  T  I  P  K  F  D  S  E  E  W  W  G  P  T  A
181     CTTAAAGGAAAGACTGACAACAGCATCAGACCCTTCAAAGTCAAATTCGATGAGGCGATGATTAAAGACCTCAAATTCCGTCTAAAGAAC
43       L  K  G  K  T  D  N  S  I  R  P  F  K  V  K  F  D  E  A  M  I  K  D  L  K  F  R  L  K  N
271     CACCGTAAGTTCACACCACCTCTCGAAGGTGTCGCCTTCGAGTACGGGTTCAACACCGCTCAGATCGACAGCTGGCTGAACTACTGGTCT
73       H  R  K  F  T  P  P  L  E  G  V  A  F  E  Y  G  F  N  T  A  Q  I  D  S  W  L  N  Y  W  S
361     GATAAGTACAACTTCGTTGAGAGGGAGGCTTTCCTTAATAAGGTCCCTCACTTCAAGACTAATATCCAAGGACTAGATATTCATTTCATA
103      D  K  Y  N  F  V  E  R  E  A  F  L  N  K  V  P  H  F  K  T  N  I  Q  G  L  D  I  H  F  I
451     CATATTAAGCCTCAGGTTCCCAAAGACGTGCAGACGGTTCCTCTCCTCATGATACACGGCTGGCCAGGCTCTGTGAGAGAGTTCTACGAG
133      H  I  K  P  Q  V  P  K  D  V  Q  T  V  P  L  L  M  I  H  G  W  P  G  S  V  R  E  F  Y  E
541     GCCATTCCTCTGCTCACGAAACAGACGCCAGGTTACAACTTCGTGTTTGAGCTGATCATCCCCAGTATACCTGGATATGGATTCTCTGAC
163      A  I  P  L  L  T  K  Q  T  P  G  Y  N  F  V  F  E  L  I  I  P  S  I  P  G  Y  G  F  S  D
631     CCTGCAGTCAGACCAGGTCTGGGCATGCCTCAAACAGCTGTCATCTTCAGGACCCTGATGAACCGTTTAGGCCACAAGAAGTTCTACGTT
193      P  A  V  R  P  G  L  G  M  P  Q  T  A  V  I  F  R  T  L  M  N  R  L  G  H  K  K  F  Y  V
721     CAGGGAGGTGACTGGGGCGCAGGTATCGTGTCGGTTATGTCCACCATCTTCCCTGAAGACATCCTGGGACATCACTCCAATATGCTTCTG
223      Q  G  G  D  W  G  A  G  I  V  S  V  M  S  T  I  F  P  E  D  I  L  G  H  H  S  N  M  L  L
811     TCTCAGCACACTTGCTCGATAATAAGATGGTTCATCGGCGCTTTCTTCCCATCCCTCATAGTCGAGGAACATCTGGCAAGCAGAATGTAC
253      S  Q  H  T  C  S  I  I  R  W  F  I  G  A  F  F  P  S  L  I  V  E  E  H  L  A  S  R  M  Y
901     CCATTGTCGACTTACTTTGCCTATGTGATGGAAGAATTCGGTTACATGCACATCCAGGCCACTAAACCTGATACTGTTGGTGTTCCTTTG
283      P  L  S  T  Y  F  A  Y  V  M  E  E  F  G  Y  M  H  I  Q  A  T  K  P  D  T  V  G  V  P  L
991     AATGATTCTCCAGCTGGTCTCTTAGCATACATCCTGGAGAAATTCTCCACATGGACTAATAAAGATAACAAGGGCAAACCTGACGGTGGA
313      N  D  S  P  A  G  L  L  A  Y  I  L  E  K  F  S  T  W  T  N  K  D  N  K  G  K  P  D  G  G
1081    CTCGCTCTGAGGTTTACTAAGGACCAACTCATTGATAACCTAATGATCTACTGGTCTACCAACTCCATTACCACTTCCATGAGGTTCTAC
343      L  A  L  R  F  T  K  D  Q  L  I  D  N  L  M  I  Y  W  S  T  N  S  I  T  T  S  M  R  F  Y
1171    GCAGAAAACTTTTCGAACAAAATAAGGGAATATAATTTGGACCAGATCACAACACAGGTCCCGACCTGGGCCCTTCAAGCGAAAAACGAG
373      A  E  N  F  S  N  K  I  R  E  Y  N  L  D  Q  I  T  T  Q  V  P  T  W  A  L  Q  A  K  N  E
1261    CTGATGTACCAACCTCCAAGCCTCTTGAACAGGAAGTACAAGAACTTGATAAACGCCACAGTCTTGGATGATGGAGGGCATTTCCTGGCG
403      L  M  Y  Q  P  P  S  L  L  N  R  K  Y  K  N  L  I  N  A  T  V  L  D  D  G  G  H  F  L  A
1351    TTCGAACTGCCCCAAGTCTTCGCTGCAGACGTGTTCAAGGCAGTGAAAGCGTTTAAAGAGTGGCATCAGACTAATCAGAAGACTGAACTG
433      F  E  L  P  Q  V  F  A  A  D  V  F  K  A  V  K  A  F  K  E  W  H  Q  T  N  Q  K  T  E  L
1441    TGATTTGGTTAGGGGTTATTAAAGTTTTTTTATGGTTTTGAAAAAAAAAAAAAAAAAAAAAAAAAAAA
463      *
```

Figure S4. Nucleotide and deduced amino acid sequence of *MsJheh*. “□” stand for residues highlighted by the box are the conserved HGWP. The positions of the primers used in the initial degenerate RT-PCR are shown by thin lines under the nucleotide sequence. The stop codon is indicated by an asterisk. The residues of the potential catalytic triads and oxyanion holes are shown.

```
HaJHE     ---------------MTSHVLALAFLLH--ACTALAWPGAASR-------SVDAYLDCGRVRGVQRSAH-GFKFASFLGVPYAKQPVGDL 65
HvJHE     ---------------MTTYVFALAFLLH--ACTALAWPGAASC-------SVRADLDSGTVCGVQRSVE-GSKYASFPGVPYAKQPLGDL 65
SlJHE     ------------MTTLAPYMFALLLLLP--ACTGHVFPSERCR--------VSARIESGTVCGVERIADGDVKYASFRGVPYAKQPLGEL 68
BmJHE     ---------MCTKFAVLLYYVIVGLVRA--YSSPAASPPSSCN--------VVAQTESGWVCGRSRRAEASTLYASFRGVPYAKQPVGEL 71
DpJHE     MRRPATETSLELPPFEMSSLVLSALVLSSLVLPSLVLPAQAVPLSDSDGCAVRAQLPGGWLCGLR----NGTSHASFLGVPYAKQPLGQL 86
MsJHE     -----MEKCGARHPDDVTTLIALAFALH--ACTALASPPSPAARCHEGIDVVPLQTDRGLVCGLRRVADNGVTYASFRGVPYAKQPVGEL 83
                              .   :       .  . *             *      * : *       .  .*** ********:*:* 21

HaJHE     RFKELEPLEHWDDVFNATSEGPICFQTDILYGRIMAPSEMS---EACIYANVHVPLHALPEFTDR-------------QRDWTP-GLPIL 138
HvJHE     RFKELEPPEHWD-VLQATNEGPICFQADVLYGRLMQKSKMS---EACIHANIHVPLRALPLGVHRPTRQLETISVPVIPEELTPPGLPIL 151
SlJHE     RFKELEPADKWD-YYYATDEGPVCYQHDVVYGRLMRPQGMS---ESCIHANIHVPMEALPEQHPD----------------VTLPGLPIL 138
BmJHE     RFKELQPAEPWTGYLDATEEGPVCYQTDVLYGSLMKPHGMN---EACIYANIHVPLYALPAAGET----------------PTKPGLPIL 142
DpJHE     RFKELQPLEPWEGFYDATSEGPICPQHDILYG-ALNPGQMD---EACMYANIHVPLHALPGYQPA---------------KTETDGLPIL 157
MsJHE     RFKELVPADPWT-VRNATQEGPICIQTDVLYKDLMNQTQMKNQDEACIYANIHVPLEPRPGVTRR--------------LAGATALLPIL 158
          ***** * : *     **.***:* * *::*   :    *.   *:*::**:***: . *                          **** 60

HaJHE     VFIHGGGFAFGSGHQDLHGPEYLVSKNAIVITFNYRLNVFGFLSMNTTKIPGNAGLRDQVTLLRWVQRNAKHFGGDPNNVTIAGQSAGAA 228
HvJHE     VFIHGGGFAFGSGHEDLHGPEYLVSRNIIVITFNYRLNVFGFLSMNTTKIPGNAGLRDQVTLLRWVQRNAKNFGGDPNDVTIAGQSAGAA 241
SlJHE     VFIHGGGFGFGSGDADLHGPEYLVSKKVIVITFNYRLNVFGFLSMNTTAIPGNAGLRDQVTLLRWVQRNAKAFGGDPREVTLAGQSAGAA 228
BmJHE     VFIHGGGFAFGSGDADLYGPEYLVTRNVVVITFNYRLNFFGFFSLDTPKVPGNNGLRDMVTLLRWVKRNARAFGGNPDNVTLAGQSAGAA 232
DpJHE     VMIHGGGFAFGSGGLGLHGPDYLMPIDVIVITFNYRLNVLGFLSLNTSYVPGNNGLRDIVTLLRWVQDNAYAFGGDPGNVTIVGQSAGAT 247
MsJHE     VFIHGGGFAFGSGDSDLHGPEYLVSKKVIVITFNYRLNVFGFLSMNTPQIPGNAGLRDQVTLLNWVQRNAKNFGGDPNDVTIAGQSAGAV 248
          *:******.****  .*:**:**:. . :*********.:**:*::*. :*** ***:****.**:  **  ***:* :**:.******. 130

HaJHE     AAHLLTLSKAAKGLFKRAILMSGTGISSFFSMSPAYAAHISNQLLFVLGIKDTDPEEIHRKLIDLPAEKLSDANAALLEQFGLTTFVPTV 318
HvJHE     AAHLLTLSKATEGLFQRAILMSGTGISSFYSSSPAFAAYMSKQLLLVLGITATDPDEIHQQLIDLPAEKLNDTNAFLLEQIGLTTFVPTV 331
SlJHE     AAHLLSLSPAAEGLFRRAILMSGTATSSFFSSSPAYAQHIKNIYLQFLGITSTDIEDIQRQLIELPAERLNDANTKLIDQMGLVTFGPTV 318
BmJHE     AAHLLTLSKATEGLVSRAILMSGAGTSTFSTTSPIFSQSINKLLFSILGVNSTNPDEIHEKLVAMPVEKLNEANRILIDQIGLTTFFPVV 322
DpJHE     SAHLLTLSTASRGLFNRAIIMSGTAISSFYTPSPMYAQLVANMFLTSVGINSTDPEEVHQRLVDLPIERIMEANKQLQNHFGLTVFTPVV 337
MsJHE     AAHLLTLSPATTGLFKRAILMSGTAASTFYSSSPAYAQLMSSYVLNILGIASTDPDHVHRKLIELPAEKLKEVNQMILDGSGLATFVPTV 338
          :****:** *: **. ***:***:. *:* : ** ::  : .  :  :*:  *: :.::.:*: :* *:: :.*  : :  **..* *.* 185

HaJHE     ESPLPGVTTIIDDDPEILIAKGRGKNIPLLVGFTSTECETFRNRLINFGFVNKIKDNPAIIIPPRVLLMTPPQLVMDLKESIERRYYNDS 408
HvJHE     ESPLPGVTTIIDDDPESLIAKGRGKNIPLLIGFTSSECETFRNRLTNFDLVKKIQDNPTIIIPPKVLFMTPPELLMDLSKTIDRKYYNGT 421
SlJHE     ESPLPGVTTIIDDDPEALLSKGRGKNIPLIVGYTNSECETFRNRFEAFDLITKIASHNFLVVPPKLLFTTPPESWSNLVSRMLGRYSDGR 408
BmJHE     ETPHPGITTILDEDPNILVQQGRGKDIPLIIGFTNSECHMFQRRFEQIDIVSKINENPAILVPSNLLYSSTLETIALVSNQISQRYSNGS 412
DpJHE     ESSFPGVTAVVDRDPELLMSQGCGAHIPLIIGFTNSECEVFRRSFQNINILERIKDNPLLSVPIKLLYGLPPQNVSAALKKIQEQYFIGE 427
MsJHE     ESPLPGVTTIIDDDPDTLIANGRGKDIPLLVGYTSAECETFRNRLEEFNLIPKIQNNSALIVPPKILFMTPIGIHADLAKEIETKYYDG- 427
          *:. **:*:::* **: *: :* * .***::*:*.:**. *:. :  :.:: :* .:  : :* .:*   .        . :  :*  .  231

HaJHE     ISIDNFVKSCSDGFYEYPALKLAQKRAETGGAPLYLYRFAYEGQSSIIKEVMELDYDGAGHIEDLTYVFRTNSVVEAPGATAPSKDDVKM 498
HvJHE     ISIDNFLKSCSDGFYEYPALKLAQKRAETGGAPLYLYRFAYEGQNSVIKAVTGLNYEGVGHIEDLTYVFRANSVVEAPDAVAPSDDDVCM 511
SlJHE     MNMDGFVKSCTEGFYEYPALKLVQKRAELDGAPVFLYQFTFESPTSIIKEVTGLSYAGAAHIEDLTYVFKANSVLEGREGAPQSLEDLRM 498
BmJHE     VDLEGFINMCTDSYYKYPAMKLAEKRSAAGDAPVFLYQFSYDG-YSVFKQAFHLHFNGAGHADDLTYVLKVN---SASGTSSSQKADDEM 498
DpJHE     PTIDEYVKYASDAYYKQPAIKVAMMRRMLRGAPAYLYEFSYHPDFSVIGQITGLNYKGAEHIEDLSFLFRAKAFEGSKGFSPLSLSDQSM 517
MsJHE     VNMNSFIKTCSAAYYEYPALKLAQQRALTGGAPVFLYRFAYEGHSNVIKQATGQTYEGVGHIEDLTYVFKPNIALNAPGATTPSNDDLSM 517
            :: ::: .: .:*: **:*:.  *    .** :**.*::.   .::       : *. * :**::::: :    .    . .  *  * 270

HaJHE     KNLMTSYFVNFMKCSKPICEDNNSLEKWPAGD---GMQYEDIVSPDMIRSKEFSSRQQDIIESFDSLTSGSQL--- 568
HvJHE     KNLMTDFFVNFMTCSKPICEDNNSLELWPAND---GMQYEDIVSPTMVRSMEFSSRQQDIIEFFDSLTSRSQL--- 581
SlJHE     KNFMTDLFVQFMSCSKPICDDKRS--LWPENN---GDQIEVIEAPGVLRTNSRSMRQLEVVDFFDNLPSRVNVV-- 567
BmJHE     KYWMTTFVTNFMRCSAPMCDETT---AWPPVTP-RELQYQDIITPNLCHQTSLTKEQLEMKNFFDKIHNGGESRLK 570
DpJHE     VHRMTTIVKNFMEDRDP----------WPPVSDGTELEYQVIDRPEVYQFSGLSKQLKEMADFFDSL--------- 574
MsJHE     KNLMTDVFVNFMKTNKPLLPS-----IWPADD---GRQFEELRSPTLILSKEFSASQQELITFFNSLASRRPPT-- 583
             **  . :**    *          **        : : :  * :      :    ::   *:.:           289
```

Figure. S5. Clustal W alignment of amino acid sequence of *M. separate* JHE with the sequences of other insect JHEs: Numbers on the right side of the alignment indicate the position of residues in the sequence of each protein. HaJHE (*H. armigera* JHE, accession number AEB77712), HvJHE (*H.viriplaca* JHE, AGB93712); SlJHE (*S.litura* JHE, ACV60229), BmJHE (*B. mori* JHE, AAR37335) and DpJHE (*D. plexippus* JHE, EHJ63342). The five catalytic motifs that are conserved in JHEs are shown in bold and thick underline in light blue background. Identical amino acid residues are indicated by asterisks, conserved substitutions by a ":" and semi-conserved sequences by a ".".

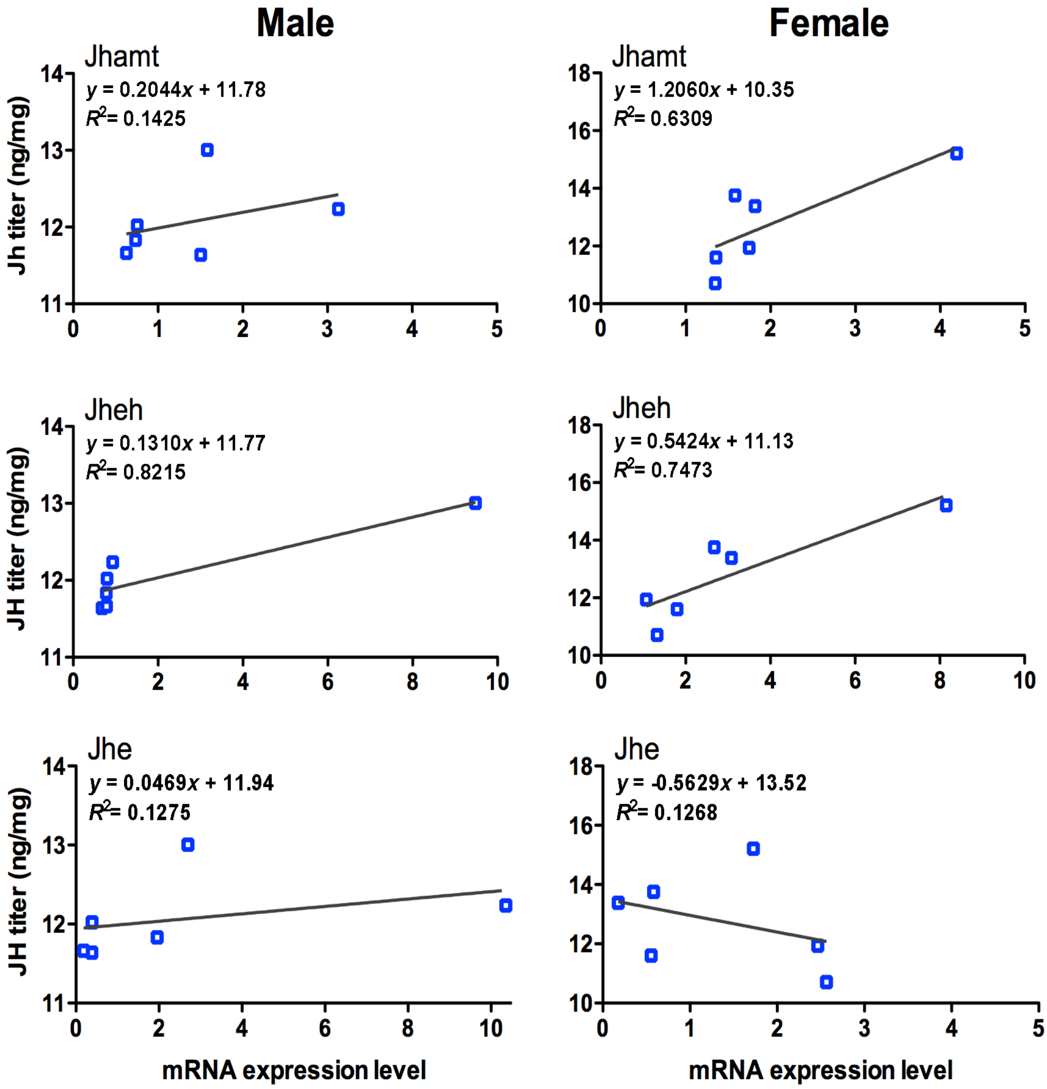

Figure S6. Linear regression analysis between monthly JH titer as the dependent variable and the transcript level of *Jhamt, Jheh* and *Jhe* normalized to the internal standard genes of β-actin and GAPDH as independent variable in *M. separata*.
